# Comparing gender discrimination and inequality in indie and traditional publishing

**DOI:** 10.1371/journal.pone.0195298

**Published:** 2018-04-09

**Authors:** Dana B. Weinberg, Adam Kapelner

**Affiliations:** 1 Queens College, CUNY, Department of Sociology, Queens, New York, United States of America; 2 Queens College, CUNY, Department of Mathematics, Queens, New York, United States of America; Indiana University Bloomington, UNITED STATES

## Abstract

In traditional publishing, female authors’ titles command nearly half (45%) the price of male authors’ and are underrepresented in more prestigious genres, and books are published by publishing houses, which determined whose books get published, subject classification, and retail price. In the last decade, the growth of digital technologies and sales platforms have enabled unprecedented numbers of authors to bypass publishers to publish and sell books. The rise of indie publishing (aka self-publishing) reflects the growth of the “gig” economy, where the influence of firms has diminished and workers are exposed more directly to external markets. Encompassing the traditional and the gig economy, the book industry illuminates how the gig economy may disrupt, replicate, or transform the gender discrimination mechanisms and inequality found in the traditional economy. In a natural experiment spanning from 2002 to 2012 and including over two million book titles, we compare discrimination mechanisms and inequality in indie and traditional publishing. We find that indie publishing, though more egalitarian, largely replicates traditional publishing’s gender discrimination patterns, showing an unequal distribution of male and female authors by genre (allocative discrimination), devaluation of genres written predominantly by female authors (valuative discrimination), and lower prices within genres for books by female authors (within-job discrimination). However, these discrimination mechanisms are associated with far less price inequality in indie, only 7%, in large part due to the smaller and lower range of prices in indie publishing compared to traditional publishing. We conclude that, with greater freedom, workers in the gig economy may be inclined to greater equality but will largely replicate existing labor market segmentation and the lower valuation of female-typical work and of female workers. Nonetheless, price setting for work may be more similar for workers in the gig economy due to market competition that will compress prices ranges.

## Introduction

Titles of traditionally published books by female authors are priced approximately 45% lower on average than those by male authors according to 2002-2012 data derived from R. R. Bowker’s *Books in Print* [1], a comprehensive bibliographic catalog used by retailers and libraries. This phenomenon parallels the workplace where women earn less than men, with a stable wage gap currently estimated at around 80 cents on the dollar [2]. The gender wage gap has narrowed considerably since the 1960s, due mostly to increasing inequality in men’s earnings rather than to gains in women’s earnings [3]. Using a natural experiment derived from the Bowker data, we examine the mechanisms behind the systematic devaluation of women’s work compared to men’s. In examining the gender price gap in publishing, this paper simultaneously draws from and contributes to our larger understanding of gender discrimination and inequality.

While the existence of the gender wage gap and the mechanisms driving it have been well-established in the literature, evolving employment relationships call for renewed investigation. In particular, the Digital Revolution has coincided with tremendous shifts in the relationship between workers and employers, giving rise since the 1980s to what researchers have identified as the “New Economy” [4]. The New Economy encompasses the “gig” economy—also referred to as the freelancer economy, platform economy, on-demand economy, crowdfunding economy, and sharing economy among other names [5, 6]—as well as other transitions from traditional employment relationships characterized by long-term jobs and internal labor markets to non-standard work arrangements characterized by greater worker mobility, greater uncertainty, and more direct influence of the external labor market. As previous research on wage inequality has tended to focus on employers’ discriminatory practices, particularly as these relate to long-term employment and internal labor markets [7, 8], the shift to the New Economy calls for greater attention to the roles of workers and external markets in producing inequality.

Encompassing both old and new forms of relationship between publishers and authors, the book industry provides a laboratory for studying various gender discrimination mechanisms that contribute to inequality. The past decade has seen an explosion in the indie (self-publishing) market. While self-publishing has long been an option for authors, the opening of Kindle Direct Publishing in 2007 concurrent with the increasing availability of portable e-reader devices provided new opportunities and incentives for authors to publish their works themselves and to use online platforms to sell their books. Other retailers like Barnes and Noble and Kobo subsequently opened their online retail platforms. In addition, the growth of the digital market for selling and consuming books provided traditional and non-traditional publishers alike the option to forego physical print runs, requiring less investment in the production and distribution of titles. These trends also allowed new publishers to enter the market and led to a proliferation of new contract arrangements between authors and publishers, with the new publishers and imprints tending to offer royalty-only contracts rather than investing upfront in advances. As a result, the number of titles published per year burgeoned as did the number of publishers and imprints, whether representative of new traditional publishers, non-traditional publishers, or indie authors.

In this paper, we examine the differences in book prices for male and female authors and follow the well-worn path of examining how much of the variance different sources of discrimination explain. To be clear, book prices do not accurately reflect author earnings. An author’s earnings are a function of advances (if received), the royalty rate, and the volume of books sold. However, book prices closely resemble output-related payment systems, like piece rates or commissions, for which a price is set, but total income depends on sales performance [9]. They resemble price setting, for example, as in the price on offer for a freelance gig in the absence of information about how many jobs will ultimately be contracted. Moreover, selling items and personal creations, including books, is an important facet of the gig economy. According to a recent Pew Research Center report, 18% of Americans earned money in 2015 by selling things online, and 24% of Americans had earned money from a digital commerce platform [10]. Finally, book prices are subject to the same mechanisms and sources of discrimination as are these payment systems, thus offering an illuminating window into discrimination mechanisms and their implications for inequality in the gig economy.

Studies of gender inequality typically attributes observed gender inequality to the behaviors and preferences of firms, as they are unable to differentiate the contributions of firms, workers, and markets each, although these are all thought to contribute. As a result, it is difficult to predict how gender discrimination might or might not manifest in the absence of influence by firms in the gig economy. Using a natural experiment in publishing, this paper compares the gender discrimination mechanisms in indie and traditional publishing to differentiate the influence of firms from those of authors and/or markets. In addition to contributing to literature on the publishing industry, this paper furthers our understanding of the dynamics of gender discrimination in the traditional and new economy through a novel exploration of how the gig economy may disrupt, replicate, or transform the gender discrimination and inequality observed in the traditional economy.

## Mechanisms driving gender inequality

In this paper, we use the framework developed by Petersen and Saporta [11] as a starting point for examining opportunities for gender discrimination and follow their approach of examining structural conditions under which discrimination is feasible rather than the motivation behind it. They identify three main mechanisms driving workplace inequality: (1) allocative discrimination, related to the differential sorting and matching of workers to opportunities and organizations that offer different pay; (2) valuative discrimination, related to a differential value placed on work characterized as typically male or female despite comparable skill and other wage-related requirements; and (3) within-job discrimination, related to differences in pay and recognition for the same job within the same organization. These discrimination mechanisms have been explored in depth in traditional firms, but not in the gig economy. In this section, we review each mechanism below both generally and in relation to traditional publishing. In the following section, we turn our attention to the potential of indie publishing and the gig economy more generally and ask, do they challenge, change, or repeat these discriminatory behaviors?

### Allocative discrimination

Through allocative discrimination, men and women occupy different industries, occupations, and jobs with different pay and reward structures. Allocative discrimination in the form of labor market segregation presents the strongest explanatory factor across studies for the gender wage gap. In a comprehensive literature review Reskin [12] explains, “[Segregation] is a fundamental process in social inequality. The characteristics on which groups are sorted symbolize dominant or subordinate status and become the basis for differential treatment. Indeed, segregation facilitates unequal treatment by subjecting groups to different reward systems” and is, furthermore, controlled and sustained by the dominant group (pages 241–242). On the employer side, gender segregation may reflect discriminatory barriers to entering particular jobs and a preference for sex-typical workers in those jobs. Male and female incumbents may be steered by employers through their hiring practices (e.g. reaching out to particular networks or selecting particular types of workers) towards or away from certain types of work [11, 12]. Allocative discrimination may also occur later in the employment relationship, at promotion into certain titles or departments or with differential dismissal [11]. On the employee side, incumbents themselves may make different human capital investments that qualify or disqualify them for particular types of jobs, or they might choose particular types of jobs based on social context and their own attitudes and perceptions [12–15]. Additionally, segregation might reflect market preferences. Reskin [12] notes that some employers justified excluding women from certain jobs based on customer discrimination, a justification that courts have subsequently ruled to be illegitimate.

In publishing, allocative discrimination is observed in the differential sorting of authors into genres based on gender. In traditional publishing, authors go through gatekeepers, namely editors and/or literary agents, to get their books published, and these gatekeepers decide which authors to publish; match authors to particular publishing imprints, which may have differences in prestige or resources; and sort their books into particular genre classifications. For example, publishers might show preferences for literary fiction by male authors or for romance novels by female authors, steering authors away from those genres when they don’t fit the gender stereotype by rejecting their books or presenting them with alternative classifications. As a result, female authors might be differentially selected for publication and highly concentrated in certain genres or publishing outlets and underrepresented in others.

### Valuative discrimination

While allocative discrimination relates to how men and women are differentially distributed in the labor market, valuative discrimination relates to the differences in value placed on comparable jobs based on whether they are done predominantly by men or women. Research on a variety of jobs and industries consistently shows that the more female an occupation, the lower the pay and prestige. Scholars have concluded that “women’s work” is devalued simply because it is largely performed by women [2, 12, 16–18], viewed as instrinsically “feminine” (e.g. because it involves nurturing or emotional work [19]), or the work tasks are viewed as ‘gender-typical’ work tasks or the working time arrangements are typically female [20]. For example, research on comparable worth in academia suggests that the wage penalty for faculty in fields with higher proportions of women could not be fully explained either by differences in the external labor market or by differences in human capital of the faculty in those fields [17].

In publishing, valuative discrimination may be observed when publishers systematically devalue female-dominated genres relative to male-dominated genres, such that books in female-dominated genres receive less investment in production and distribution and have attendantly lower prices and prestige.

### Within-job discrimination

Even after controlling for gender segregation and the lower value to work predominantly done by women, in studies of wage inequality a small gender gap in earnings remains, reflecting within-job discrimination. Researchers have attributed this difference to more direct discrimination related to biased evaluations of workers themselves, whether at the time of hire or in subsequent evaluations. Overall, women tend to receive less favorable ratings than male workers, although greater representation of female managers in relatively high-status positions reduces the gender wage gap [18]. In cultural industries, work by female artists receives fewer reviews or accolades, compared to male artists in the same genre [21]. In academia, seemingly objective constructions and evaluations of excellence incorporate gender inequalities, whether by applying standards that are less likely to be met by less privileged groups or less likely to be attributed to members of those groups by evaluators. The evaluation gap is likely due to gender differences in self-presentation or due to evaluators’ gender-based biases [22], following both a “Matthew effect” [23] of cumulative advantage and a “Matilda effect” [24] of systematic underestimation and minimization that leads to cumulative disadvantage. These patterns are reflected in scholarly authorship, where from an achievement perspective men and women might be equal in raw number of publications but women accrue less prestige and recognition [25]. Of the different types of discrimination, Petersen and Saporta [11] note that within-job discrimination is likely the least important in explaining overall wage inequality, particularly as numerous legal protections create strong disincentives to knowingly proliferate this type of discrimination within a given firm.

In the publishing industry, within-job discrimination would appear as within-genre differences in book prices based on author gender, with books by female authors priced lower than those by male authors even within the same genre and format. One might argue that lower prices might encourage more sales, but a gender-based differential would suggest that publishers perceive that the books by female authors have a lower market value than those by male authors such that a lower price is needed to encourage sales to the same anticipated audience.

## Discrimination in the gig economy

This paper investigates gender discrimination and inequality in traditional and indie publishing related to author selection into genres (allocative discrimination), differences in prices by genre based on gender composition (valuative discrimination), and differences in book prices for male and female authors publishing books in the same genres and formats (within-job discrimination). While novel and interesting in its own right, this exploration also has more general implications for understanding how gender discrimination operates in the gig economy compared to the traditional economy.

Exemplifying the gig or platform-mediated economy, indie publishing has democratized the book industry. In the past publishers and associated gatekeepers determined the cultural value of a given book, set the price, and decided what would and would not be published. Now, anyone can publish and market a book. In indie publishing, authors choose for themselves genre classifications for their books, how much to invest in production and distribution, and what prices to set, albeit with some influence from the sales platforms which have rules about acceptable content, provide the menu of genre categories from which to choose, and in the case of Amazon’s Kindle Direct Publishing influence pricing through price-based royalty incentives. As an alternative venue for publishing, there is a certain amount of stigma still attached to indie publishing, but this stigma has diminished as well-known authors have chosen to go the indie route with both their backlists and new works and as success stories have proliferated.

If publishers have been the architects of much of the discrimination in the publishing industry, then we might hypothesize that this newfound independence of authors will create greater gender equality. More generally, as the gig economy puts power in the hands of workers, we might expect to see disruption of traditional patterns of discrimination and gender inequality.

Alternatively, one might argue that publishers’ (or even platforms’) discriminatory behaviors reflect the biases and prejudices of the broader client market. Following Zukin and MaGuire [26], we might view publishers as active participants in creation of consumer culture around their books, seeing them as part of an institutional field or “set of interconnected economic and cultural institutions centered on the production of commodities for individual demand” (p. 175, see also [27, 28]). As such, their decisions about book pricing may reflect external forces related to audience preferences and demand. (In some cases, publishers have implemented intentional strategies to create markets with attendant signals based on book pricing, production, and distribution, as in Radway’s [27] description of the romance paperback market.) As indie authors also face these same market pressures, then indie publishing is likely to replicate rather than reject the gender disparities observed in traditional publishing. To the extent that market preferences, rather than firm preferences, drive the discrimination mechanisms in the traditional economy, then gender disparities in the traditional economy will be replicated in the gig economy as workers more directly face the market.

Finally, the changing nature of employment in the gig economy provides new discrimination opportunities, particularly when employment relations between “workers” and “clients” are mediated by platforms or placement agencies that are not technically employers. For example, Fernandez-Mateo [8] found that female contractors receive both lower rates for their contracts and a lower volume of work. We might, therefore, anticipate disparities to be even greater in indie publishing than in traditional publishing, as indie authors approach the market without the buffer of publishing firms. More generally, if the discriminatory preferences are pronounced in the external market or on the part of platforms, then we might see an even greater trend toward inequality in the gig economy than in the traditional economy as behaviors may be less easily observed and regulated, providing greater opportunity to discriminate [6, 11].

## Methods

The growth of indie publishing creates the opportunity for a natural experiment that allows us to differentiate between the behaviors of publishers and authors or markets in contributing to gender discrimination and inequality in the book industry. Through this example, we test the conflicting expectations regarding gender discrimination in the gig economy, namely whether indie publishing will remove, replicate, or increase the gender-based labor market segregation, devaluation of work done primarily by women, and gender differences in pay within jobs observed in the traditional economy.

Queens College-CUNY IRB reviewed and exempted this study as human subjects research.

### Data

Indie authors publish titles on their own or else through a vanity publisher or platform facilitator such as Smashwords, Amazon’s CreateSpace or Kindle Direct Publishing. The key distinction between traditionally-published and indie-published titles is whether the author directly bears the cost of publishing. Using R. R. Bowker’s *Books in Print* from 2002-2012, a catalogue of all books published in North America with ISBN codes, we compare gender-based allocation to subject categories, valuation of genres, and prices for titles published by traditional publishers and by indie authors. Parentheticall, note that it is possible to publish ebooks through Kindle Direct Publishing without an ISBN, and some authors choose to do so to avoid the expense of the ISBN. These digital-only books are not included in Bowker’s catalogue unless they also were published in another format or on another platform with an ISBN. Note that these non-ISBN titles are only available on Amazon.

*Books in Print* has over 11.5 million books in its catalog for this period. Book metadata in *Books in Print* are entered by the book publisher or, in the case of indie publishing, by the author. The same title might be represented by several different book entries if published in multiple formats across different years. We aggregated entries by author and title and retained the maximum price, formats in which the book was published, first publisher, and earliest publication date. Aggregating from books to titles yielded a total of *n* = 2, 456, 510 titles in the dataset.

#### Variables

Publishers were identified in several ways. First, we identified the largest publishing houses, the Big Five and large publishers, using a list of publishers and imprints from Publishers Marketplace. We also used lists of small and independent publishers and university presses to match to the publishers on our list. We then hand-coded any unmatched publisher with 1,000 or more ISBNs as well as those with the words with names indicating they might be colleges or universities. Remaining publishers with words like “association” or “institute” were identified as institutional presses. These efforts identified 13,800 traditional publishers and 86 self-publishing companies. Of the remaining unmatched publishers, 92,589 (76.5% of publishers) had published titles by fewer than five authors between 2002 and 2012 and were coded as indie authors. Note that we used five as the cutoff since authors sometimes write under multiple pen names but will use the same publisher name for all of them. Vanity presses and other self-publishing companies were also coded as indie authors. The remaining 12.1% of the publishers in the data were unmatched. These may represent small presses or imprints, and as a conservative measure we include them among traditional publishers.

**Author Gender** In this study, author gender is derived from the gender of the author’s name. To determine the gender of author names, we first retained only single-author titles. 14.4% of the titles were excluded because they either had multiple authors or were not in the prescribed format of “last name, first name” with up to two middle names or initials. Excluding the “ineligible” cases drops the sample from 2,456,510 to 2,103,601. The percentage of such cases is significantly higher in traditional publishing 15.6% than in indie 5.5%.

We matched first and middle names to lists of male and female baby names from different regions or languages that represented the most popular countries of origin from the 2010 US Census. This resulted in a collection of unique names hailing from 48 countries. First and middle names were used to identify author names as male, female, androgynous, or unmatched. Initials and names that appeared on both male and female lists were coded as androgynous. For authors with one name that was androgynous or unmatched, the gender(s) of the remaining names were used to identify gender.

Names that appeared on both (or neither) male and female lists were considered androgynous. The gender of a name is not necessarily an accurate reflection of author gender, particularly because some authors write with pen names. For example, a famous female author appears as male under the pseudonym Robert Galbraith and as gender-unknown when published as J. K. Rowling. Socially, the use of androgynous names has remained relatively stable over time, but the use of such names has increased among parents of daughters [29]. Therefore, it is possible that use of androgynous pen names is more common among female authors than among male authors based on these same trends in taste. Finally, we see the perceived gender of the pen name as a conscious choice in relation to the market, akin to construction of appearance in social relations. As Ridgeway and Correll [14] state, “Knowing that they will be categorized in this way, most people carefully construct their appearance according to cultural gender rules to ensure that others reliably categorize them as belonging to the sex category they claim for themselves” (p. 515). In traditional publishing, the choice of what name to put on the cover may be influenced by the publisher as well as by the author.

**Investment and Prices** Book prices reflect publishers’ or indie authors’ judgments both about a book’s economic value as an immediate consumer product and its perennial cultural value, a judgment of what will “survive the test of time” [30, 31]. Book prices encompass both of these determinations. Prices reflect decisions about how much to invest in production, distribution, and marketing as well as expectations about the audience for a book. Book format in a strong determinant of price, as format relates to the production and distribution costs that must be covered in order to make a profit. For example, the materials for hardcover books are more expensive than for softcover or e-books, and their greater bulk and weight make them more expensive to distribute. A single title may be produced in multiple formats, but not all titles will be selected for production in more expensive formats. For example, hardcover books are typically perceived as having greater prestige and cultural value. Books published in hardcover format are expected to have longer staying value than those published only as paperbacks or ebooks. E-books have the lowest production and distribution costs. Not all titles receive the investment related to production in more expensive formats.

To measure price, we use the highest retail price across book entries for each title.

We also control for investments in a given title based on the formats in which that title appeared. Based on the binding codes supplied by publishers and authors, we ascertained whether a title was ever published in one of these most popular book formats: hardcover, trade paperback, mass market paperback, ebook, or audiobook. We then created an ordinal variable for investment, reflecting the most expensive format in which a book was published: ebook = 1, mass market paperback = 2, trade paperback = 3, and hardcover = 4. Since audiobooks were sometimes but not consistently priced higher than other formats, we used a separate dummy variable to indicate whether the title was ever published as an audiobook.

**Genre** BISAC codes reflect the subject of a given book. Publishers and indie authors may list their books as having more than one of these detailed subject codes in their ISBN metadata. For this study, we used the category heading for the *first* BISAC code in the earliest entry for a given title. The BISAC is a fine-grain measure, and there are a total of 3,556 BISAC codes in the data. We collapsed these codes using the coarser-grain category heading with the exception of the fiction category heading. Since the fiction category heading contains the largest number of titles by far, we further subdivided fiction into its nineteen subcategories. In total, we considered 70 genres.

### Analysis

Our natural experiment compares traditional publishing with indie publishing in terms of: (1) the differential sorting of authors into genres (allocative discrimination), (2) the between-genre differences in the value placed on books as these relate to the gender distribution of authors (valuative discrimination), and (3) the gender differences in book prices within genres (within-job discrimination).

To investigate allocative discrimination, we first examine the differences in the allocation of titles by male and female authors to each genre. Given the presence of a third author name category, where the author gender is unknown whether because the name is androgynous or because we could not match it, we provide separate analyses using the proportion of male and female authors respectively. We calculate z-scores for the proportion differences between indie and traditional publishing for each genre. Next, we consider the case of gender parity in which each genre would have the same percentage of titles by female or male authors based on their respective representation overall. Using these gender means for each case, we create “parity-adjusted” distribution, examining the deviation from the egalitarian mean both by genre and overall. Finally, we compare the distance between these parity-adjusted distributions for traditional and indie publishing to determine the difference in the level of gender segregation relative to an egalitarian standard, using both the average absolute value of the differences by genre (norm 1) and the average root mean-squared error (RMSE) (norm 2).

To investigate valuative discrimination, we examine the relationship between the percentage of titles by female and male authors in a genre and log price. Comparing traditional and indie publishing side by side, we regress log price on the percentage of male or female titles in each genre using a hierarchical linear model of titles nested within genres. Note that similar results were obtained using a combined model of indie and traditional publishing for each gender case, which confirmed a statistically significant difference in the interaction between gender percentage and indie publishing. We decided to employ separate models in presentation due to the greater ease in interpretation. We use the xtreg procedure in STATA to fit between effects models; this allows for a comparison of book prices between genres in relation to the predominance of titles by male or female authors.

Finally, we examine the gender gap in prices for titles by male and female authors using a variety of models. We again compare traditional and indie publishing side-by-side in separate regressions of log price. Note that similar results were obtained using a combined model and showing a significant interaction for author gender with indie publishing. Again, we decided to employ separate models due to the greater ease in interpretation. We start with an ordinary least squares (OLS) regression. We then introduce book genre as a fixed effect using the xtreg procedure in STATA; this allows for a view of gender inequality within genres. We then augment the model with additional covariates from Table 1. For author gender, the comparison group is male, and dummy variables for female and unknown gender are included. In the traditional publisher models, Big Five publishers, the five largest publishing houses in the US book market, are the comparison group. For the genre categories, general fiction serves as the comparison category, and we control for year using dummy variables for 2002-2012 where 2002 serves as the base category.

**Table 1:** Summary statistics. The *book price* refers to the maximum price aggregated over all formats. *Investment* is an ordinal metric where 0 indicates ebook, 1 indicates mass market paperback, 2 indicates trade paperback, and 3 indicates hardcover. The rest of the variables are binary.

	Female Author Name		Male Author Name		Gender Unknown Author Name	
Variable	# of Titles	avg. ± s.e. [min, max]	# of Titles	avg. ± s.e. [min, max]	# of Titles	avg. ± s.e. [min, max]
Book Price	329,068	$37.45 ± $137.05 [$0.01, $38,515.00]	546,288	$55.37 ±$156.11 [$0.01, $29,095]	370,883	$81.82 ± $244.70 [$0.01, $54,009]
Investment	467,438	1.73 ± 1.18 [0, 3]	814,019	1.82± 1.16 [0, 3]	516,958	1.76 ± 1.16 [0, 3]
Published as Audiobook	545,757	3%	946,426	3%	607,866	5%
Big Five Publisher	546,711	4%	948,269	3%	608,621	3%
Large Publisher	546,711	4%	948,269	2%	608,621	2%
Academic Publisher	546,711	7%	948,269	9%	608,621	7%
Institutional Publisher	546,711	2%	948,269	2%	608,621	2%
Traditional Publisher	546,711	51%	948,269	52%	608,621	54%
Audiobook Publisher	546,711	1%	948,269	1%	608,621	1%
Indie Author	546,711	13%	948,269	13%	608,621	14%
Unmatched Publisher	546,711	18%	948,269	18%	608,621	18%
First published in 2002	546,711	6%	948,269	6%	608,621	5%
First published in 2003	546,711	6%	948,269	7%	608,621	6%
First published in 2004	546,711	7%	948,269	7%	608,621	6%
First published in 2005	546,711	7%	948,269	8%	608,621	7%
First published in 2006	546,711	8%	948,269	8%	608,621	8%
First published in 2007	546,711	8%	948,269	8%	608,621	8%
First published in 2008	546,711	9%	948,269	9%	608,621	13%
First published in 2009	546,711	11%	948,269	10%	608,621	11%
First published in 2010	546,711	12%	948,269	12%	608,621	12%
First published in 2011	546,711	14%	948,269	13%	608,621	13%
First published in 2012	546,711	13%	948,269	11%	608,621	12%

This modeling approach follows the tradition in much inequality research of first examining unconditional differences in wage means and then attempting to reduce that difference with putative confounders [3]. In addition to differences in means, we wish to understand whether the price gap behaves differently at the top and bottom of the price distribution i.e. inequality across the distribution of book prices. We compare the within-genre prices for books by male and female authors at various quantiles via unconditional quantile regression [32]. Specifically, we use the xtrifreg procedure in STATA [33] to fit a quantile fixed effects model of log price where genre is the fixed effect. These models use listwise deletion for entries with missing data, reducing the sample in these models to 721,431 cases.

#### Missing data issues

The key variables of price and genre introduce significant sources of missing data. Of the eligible titles, 69.8% reported the BISAC codes from which we derived genre categories, while only 59% had retail price reported in their metadata. We did not use multiple imputation to correct for missing data since the dataset had so few available variables. The regression models use listwise deletion for cases with missing variables. For the regression analyses, our sample for analysis includes between 862,533 and 1,246,239 of the eligible single-authored titles, or between 34% and 59% of cases (N’s are noted in the tables).

Analysis of the missing data suggests significant differences by year, publisher type, and gender. Compared to 2002, the odds of having missing data increase every year, with the biggest increases starting in 2009 as more digital titles hit the market. Surprisingly, with the exception of audiobook publishers, Big 5 publishers’ titles are the most likely to have missing data, with university publishers and indie authors the least. Investment is negatively associated with exclusion, such that works that receive higher investments from publishers are more likely to be included in the analysis. Finally, while gender does significantly relate to the odds of having missing data, female author names are slightly less likely to be excluded from the analysis sample (*OR* = 0.96, *p* = 0.000) while author names with unknown genders are more likely to be excluded (*OR* = 1.13, *p* = 0.000). In terms of what this means for our findings, female authors may be slightly overrepresented in the analysis compared to their actual representation in the catalog while lower priced books are less likely to be included. Thus, any findings of differences in cultural valuation of works by male and female authors are likely to err on the conservative side.

## Results

Titles by authors with identifiably female names comprise 26% of single-author titles in *Books in Print*. Titles by authors with identifiably male names account for 45% of the titles, and the remaining 29% of single-author titles are by authors with names of indeterminate gender, whether because they use initials, have androgynous names, or have names that did not appear in the baby names databases. On average, titles by female authors are priced $17.92 lower than those by male authors (*p* = 0.000), with an average price of $37.45 compared to $55.37. Overall, books by authors with female names also receive slightly less investment on average from publishers (*p* = 0.000), meaning that they are less likely to be published in the formats that are more expensive to produce and distribute. Investment is highly related but not collinear with price, with a Spearman’s rank correlation of 0.45 (*p* = 0.000).

### Allocative discrimination

In both traditional and indie publishing, there is substantial gender-based sorting of authors. Both types of publishing sort authors into genres, but in traditional publishing there is also an additional pathway for allocative discrimination related to publisher type.

We find an association within traditional publishing between gender and publisher type (*χ*^2^ = 1.7+e04, p = 0.000). Relative to their overall representation in the catalog, titles by female authors are overrepresented among the largest publishers, namely the Big Five and other Large Publishers, and also among Audiobook Publishers. They are underrepresented relative to male authors among Academic and University Presses, Institutional Publishers, and other Traditional Publishers.

In the sample, titles by female authors are slightly more likely to be indie published than traditionally published (13.24% compared to 12.98%). However, we refrain from drawing conclusions about whether works by female authors are more likely to be indie published given the substantial number of indie titles potentially published outside of the data captured by Books in Print. The hierarchy of publishers adds an additional layer to the allocative discrimination story in traditional publishing, but this pathway is absent in indie publishing.

Table 2 details the distribution of titles based on the gender of authors’ names. Comparing the raw differences in proportion between traditional and indie publishing, we find significant differences in allocation for many genres, suggesting a different distribution by gender. Is indie more or less egalitarian than traditional publishing?

**Table 2:** Gender distribution and average maximum price per book by genre.

	Traditional Publishers						Indie Authors					
Subject Category	# of Titles	Author Gender (%)		# Titles w/ Retail	Max Price ($)		# of Titles	Author Gender (%)		# Titles w/ Retail	Max Price ($)	
		F	M	Price	Avg.	s.e.		F	M	Price	Avg.	s.e.
Antiques & Collectibles	2629	24	57	1134	44.60	69.62	355	20	59	259	42.33	44.52
Architecture	9165	23	50	3260	62.39	86.69	584	22	53	325	36.43	48.30
Art	21705	30	44	7853	57.09	167.64	2622	28	46	1542	52.87	343.63
Bibles	587	16	60	434	67.62	437.71	62	16	55	43	23.16	12.16
Biography & Autobiography	41557	25	51***	22045	37.30	71.58	8423	30***	41	6591	21.06	33.39
Business	79864	16	58	45307	83.33	215.77	8061	18***	57	6288	33.68	93.78
Comics & Graphic Novels	14434	9	56	2151	14.80	19.66	905	12**	60***	763	23.59	253.09
Cooking	13232	47	26	5586	28.41	46.15	2420	46	25	1898	22.37	58.17
Computers	38539	12	58	23370	94.20	146.23	1414	14	63***	1113	59.60	226.90
Crafts & Hobbies	7335	56***	17***	3245	33.69	242.72	742	50	25	588	27.49	58.45
Design	4055	36	36	1372	54.57	107.72	215	32	43*	120	34.54	27.23
Drama	7031	22	54	4343	15.70	28.18	626	24	51	490	24.14	159.75
Education	37816	37***	41	19959	56.77	113.68	2505	33	42	1940	41.92	278.48
Family & Relationships	17300	42	33***	10488	27.47	61.11	5072	41	30	4215	17.46	31.87
Fiction	143563	35***	36	92739	18.14	35.12	32155	24	45***	27828	16.67	16.72
General	19965	33***	38	11647	21.88	191.83	6207	25	44***	102277	16.62	98.35
Action & Adventure	6043	15	53	4653	18.82	43.50	2313	16	53	5256	16.62	15.96
Racial or Ethnic	810	39**	29	320	23.07	15.72	115	26	44**	2007	17.55	8.19
Anthologies	707	31	41	339	22.09	20.60	94	23	41	98	18.69	6.41
Christian	2254	46***	25	1368	17.70	18.95	737	27	43***	77	18.90	16.26
Contemporary Women	293	57	9	174	18.80	7.09	80	51	19	672	17.41	7.46
Crime	165	16	55	94	20.07	7.53	54	15	56	69	18.96	6.36
Erotica	6474	49***	16	4974	7.84	7.37	202	35	24*	43	14.45	7.50
Fantasy	8827	30***	41	5863	18.21	8.80	2104	26	41	174	16.36	13.21
Historical	7405	29	44	4604	22.60	54.15	2144	24	47	1852	19.05	10.36
Horror	3292	17	55	2422	17.72	44.28	851	17	54	1836	15.37	19.77
Legal	362	23	53	184	22.97	35.30	91	20	58	750	18.69	12.61
Literary	413	15	53	143	28.46	14.93	12	33	42	77	16.41	7.03
Mystery & Detective	15410	33***	41	9427	25.35	42.66	2548	23	48***	7	17.06	4.46
Romance	30025	60***	10	20805	13.65	51.04	3965	41	23***	2239	15.14	14.39
Science Fiction	8654	16**	57	5730	17.15	17.51	2435	14	57	3466	16.96	8.48
Short Stories	5354	27***	44	3277	15.81	30.77	1620	21	49***	2137	15.53	41.55
Thrillers	7825	20***	52	5116	18.06	12.23	2442	15	56***	1389	16.35	14.93
Other	19089	28***	43	11466	19.76	9.99	4088	22	48***	2121	17.16	10.04
Foreign Language	10019	29*	36	5858	61.59	43.62	462	24	40*	249	29.02	7.76
Games	6478	12	65	3210	25.68	8.71	956	11	63	811	20.40	7.75
Gardening	2898	34	42	947	39.63	56.10	356	32	43	262	24.57	21.78
Health & Fitness	15915	34	40*	9479	46.09	141.65	4035	37**	38	3279	23.30	30.05
History	54915	16	60***	27426	52.05	128.03	5029	18*	56	3354	25.90	55.09
House & Home	1637	32*	42	710	41.43	52.65	172	24	53**	125	26.50	23.14
Humor	5655	19	54	2854	17.41	33.29	2253	19	53	1943	15.19	19.26
Juvenile Nonfiction	61689	50***	28	40201	27.04	117.41	2669	41	32***	2103	17.78	31.20
Juvenile Fiction	58130	46	27*	33354	15.79	30.88	9376	44	26	7851	14.80	20.96
Language Arts & Disciplines	23455	34	41*	11885	68.62	110.50	1299	31	38	831	25.89	35.38
Law	28817	18*	58	15227	104.67	131.11	1479	16	56	922	37.88	69.67
Literary Collections	4643	26	47**	2559	39.51	147.62	1238	27	43	987	17.88	26.72
Literary Criticism	23281	31***	46	11959	70.98	155.76	652	23	44	289	25.12	24.50
Mathematics	19387	11	52	13073	98.80	113.72	470	15**	53	340	30.49	36.82
Medical	48684	24	49***	31229	113.10	207.08	2703	29***	46	2065	49.28	264.21
Music	19311	17	60***	11939	32.72	95.90	1974	19	54	1520	25.22	33.36
Nature	11484	20	53***	5944	60.53	84.37	1387	26***	49	996	24.06	50.71
Non-Classifiable	1158	42***	31	650	45.43	77.46	210	29	26	186	19.90	10.58
Body, Mind, & Spirit	15221	28	45***	10469	21.23	50.32	5171	31***	39	4336	18.21	49.71
Performing Arts	11894	24	55***	5445	45.65	74.54	852	23	49	639	27.71	55.85
Pets	3055	45	25	1909	24.46	27.92	589	46	30*	485	18.17	13.34
Philosophy	13838	13	61*	7271	73.80	89.94	1036	12	59	803	21.09	19.18
Photography	5832	18	60*	2189	97.17	764.61	1040	20*	57	807	52.42	150.75
Poetry	33149	32	42	19417	17.54	30.81	10040	31*	39***	8321	16.55	39.40
Political Science	46400	16***	56	20854	71.57	81.83	2353	11	58*	1460	21.97	34.14
Psychology	24236	29	50***	12934	61.26	109.45	2013	29	45	1576	22.24	25.82
Reference	12577	29	47	7835	60.63	221.67	2622	28	46	1951	33.44	64.90
Religion	76103	17	58***	50845	32.40	96.90	13344	22***	49	10872	17.87	40.01
Science	53261	11	52	33870	167.27	548.13	1676	13*	59***	1108	39.22	85.53
Self-Help	14968	30	44***	10112	24.23	55.91	7338	31*	40	6224	18.95	29.35
Social Science	73816	27***	48	34166	62.65	102.83	4692	22	48	3002	25.63	138.34
Sports & Recreation	16015	10	66*	7416	30.50	65.51	2541	11*	65	2000	25.28	69.29
Study Aids	1522	40***	47	1001	30.39	30.10	70	13	53	53	40.26	42.30
Technology & Engineering	38469	8	54	22535	146.03	291.90	1538	13***	60***	1026	45.42	101.09
Transportation	7935	7	70***	3309	60.79	105.74	941	11***	65	666	40.62	139.08
True Crime	813	20	61***	303	38.51	44.92	67	25	43	53	22.94	16.45
Travel	18636	26***	50	8778	28.97	91.51	3434	23	48	2607	25.40	195.97
Total	1304108	26	47***	732448	57.56	180.34	164238	27***	45	130105	22.55	87.59

*** *p* < 0.001, ** *p* < 0.01 and * *p* < 0.05 denotes statistical significance of difference of the proportion of author gender comparing traditional publishers to indie authors. The notation appears in the column where there is a positive difference.

**Fig 1:**
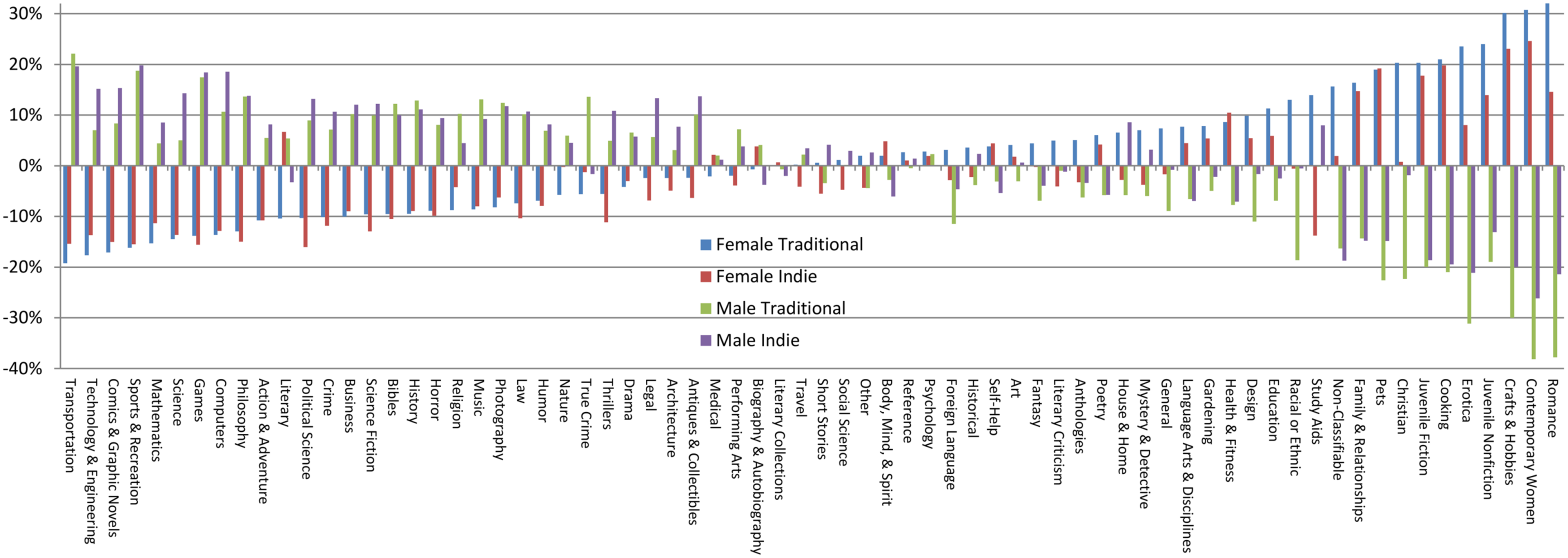
Parity-adjusted distribution by genre.

Overall, male authors outnumber female authors in both traditional and indie publishing in similar proportion. Titles by female authors comprise 25.9% of traditional titles and 26.65% of indie titles, while titles by males comprise 47.45% of traditional and 44.9% of indie titles. (Recall that the there is also a third gender category when the author gender is unknown.) In an egalitarian allocation to genres, the distribution of female and male titles in every genre would reflect these overall percentages. Fig 1 shows the distance from parity, or the deviation from these respective gender means, for each genre in traditional and indie publishing. At a glance, the distribution of titles by male and female authors across genres follow similar patterns for both types of publishing, suggesting an overall replication of the traditional pattern in indie.

**Fig 2:**
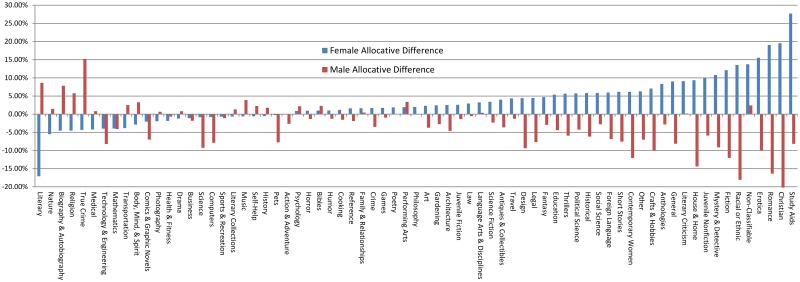
Comparison of parity-adjusted distributions for traditional vs. indie publishing.

Digging deeper, we show the difference by genre for these parity-adjusted distributions for traditional compared to indie in Fig 2, showing how much further from equality traditional is than indie for each genre. While we find differences in representation genre by genre, our measures of distance between the traditional and indie parity-adjusted distributions are relatively small. Norm 1, the absolute value of the differences averaged across genres, is only 4.99% for titles by male and 5.03% for titles by female authors, respectively. In other words, compared to a standard of parity, the difference for traditional publishing compared to indie is only plus or minus approximately 5% on average across genres. Norm 2, the RMSE across genres, is 0.87% for females and 0.80% for males, also a relatively small difference. Together, these results suggest less allocative discrimination in indie publishing overall, but not enough to signal disruption.

It seems that indie publishing exhibits a slightly less concentrated version of the gender segregation observed in traditional publishing.

### Valuative discrimination

**Fig 3:**
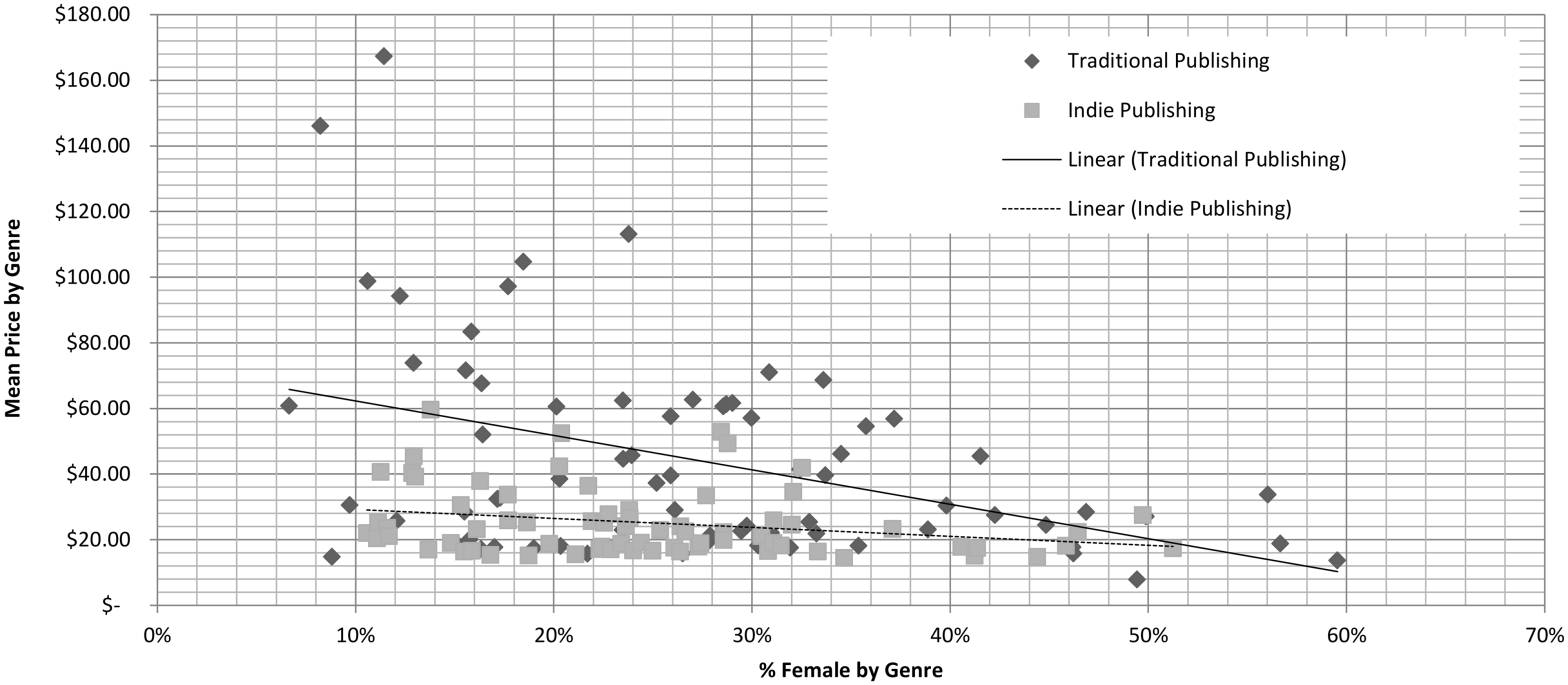
Mean price by genre versus degree of female authorship for both indie and traditional publishing. Linear trends included.

We compare the prices of different genres based on their proportions of titles by male and female authors. Fig 3 shows the relationship between price and the predominance of titles by female authors. We observe a negative, linear relationship in both traditional and indie publishing between prices and the concentration of titles by female authors. To test this observation, we used hierarchical linear regression models comparing the prices between genres based on the percentage of titles by female authors. For traditional publishing, as female authorship increases by 10%, price decreases by about 16.5% but only by 5.5% in indie publishing. Conversely, using the same approach to model the effects of male predominance, we find that as the proportion of male authorship increases by 10%, prices increase by 14.7% in traditional publishing and by 7.7% in indie publishing. All effects reported here are significant at a *p* < 0.001 level.

Together, these findings demonstrate valuative discrimination: female-dominated genres are priced lower than male-dominated ones. However, these differences are less extreme in indie publishing, where prices across genres are more similar and prices are compressed into a smaller range.

### Within-job discrimination

Using OLS (Model 1, Table 3), we find that titles by female authors are priced significantly lower than those by male authors. However, there is a striking contrast in the size of the difference, 45% (*p* = 0.000) among traditional publishers and 7% among indie (*p* = 0.000) (100 × the coefficients represent percent differences in price). This first model considers the overall difference in prices based on authors’ gender and does not distinguish between any of the discrimination mechanisms. Note that gender itself explains very little of the variation in log price (about 3% for traditional and almost none for indie) at the level of an individual title.

**Table 3:** Natural experiment regression model results for log maximum price. Coefficients × 100 can be interpreted as percent changes in price.

	Model 1: OLS						Model 2: Fixed Effects						Model 3: Fixed Effects with Covariates					
	Traditional Publisher			Indie			Traditional Publisher			Indie			Traditional Publisher			Indie		
	$\hat{\beta }$	$s_{\hat{\beta }}$	*p*_val_	$\hat{\beta }$	$s_{\hat{\beta }}$	*p*_val_	$\hat{\beta }$	$s_{\hat{\beta }}$	*p*_val_	$\hat{\beta }$	$s_{\hat{\beta }}$	*p*_val_	$\hat{\beta }$	$s_{\hat{\beta }}$	*p*_val_	$\hat{\beta }$	$s_{\hat{\beta }}$	*p*_val_
Constant	3.57	0.00	0.00	2.42	0.00	0.00	3.42	0.00	0.00	2.82	0.00	0.00	2.39	0.01	0.00	1.94	0.01	0.00
Female																		
Gender																		
Name	-0.45	0.00	0.00	-0.07	0.01	0.00	-0.09	0.00	0.00	-0.05	0.00	0.00	-0.09	0.00	0.00	-0.04	0.00	0.00
Unknown																		
Gender																		
Name	-0.07	0.00	0.00	-0.04	0.01	0.00	0.05	0.00	0.00	-0.02	0.01	0.00	0.05	0.00	0.00	-0.02	0.00	0.00
Audiobook																		
Format													0.84	0.01	0.00	0.50	0.06	0.00
Investment													0.32	0.00	0.00	0.41	0.00	0.00
Large																		
Publisher													0.06	0.01	0.00			
Academic																		
Publisher													0.40	0.01	0.00			
Institutional																		
Publisher													0.07	0.01	0.00			
Traditional																		
Publisher													0.45	0.01	0.00			
Audiobook																		
Publisher													0.59	0.03	0.00			
Unmatched																		
Publisher													-0.03	0.01	0.00			
2003													-0.02	0.00	0.00	0.01	0.01	0.19
2004													0.02	0.00	0.00	0.01	0.01	0.27
2005													0.10	0.00	0.00	0.01	0.01	0.24
2006													0.15	0.00	0.00	0.04	0.01	0.00
2007													0.13	0.00	0.00	0.12	0.01	0.00
2008													0.07	0.00	0.00	0.11	0.01	0.00
2009													0.05	0.00	0.00	0.12	0.01	0.00
2010													0.12	0.00	0.00	0.09	0.01	0.00
2011													0.03	0.00	0.00	0.07	0.01	0.00
2012													0.00	0.00	0.56	-0.02	0.01	0.06
*σ*_*u*_							0.57			0.24			0.49			0.21		
*σ*_*e*_							0.85			0.61			0.75			0.51		
*ρ*							0.31			0.14			0.30			0.14		
# of Titles	1035818			210421			732448			130105			618646			102757		
*R*^2^ overall	0.03			0.00			0.02			0.00			0.25			0.28		
*R*^2^ within							0.00			0.00			0.21			0.29		
*R*^2^ between							0.14			0.06			0.63			0.34		

Switching to a fixed effects model (Model 2, Table 3) takes into account the genre-level effects of pricing. Essentially, it separates out the allocative discrimination attached to the gender-related distribution of titles across genres and the valuative discrimination reflected in gender-related pricing of those genres and displays the within-genre differences in prices. In Model 2, the female price gap decreases to 9% (*p* = 0.000) for traditional publishers and 5% (*p* = 0.000) for indie. Adding investment by publisher, audiobook publication, publisher type, and year of publication to the model reveals strong associations with book pricing (Model 3, Table 3). Upon addition, the female price penalty in traditional publishing is unchanged but for indie, the price gap is reduced to 4%. Furthermore, this information enhances the explanatory power of the models, such that overall they explain 25% of the variation in log prices among traditional publishers and 28% among indie authors, with the greatest explanatory power seen between genres, 63% and 34% of the log price variation, respectively.

**Fig 4:**
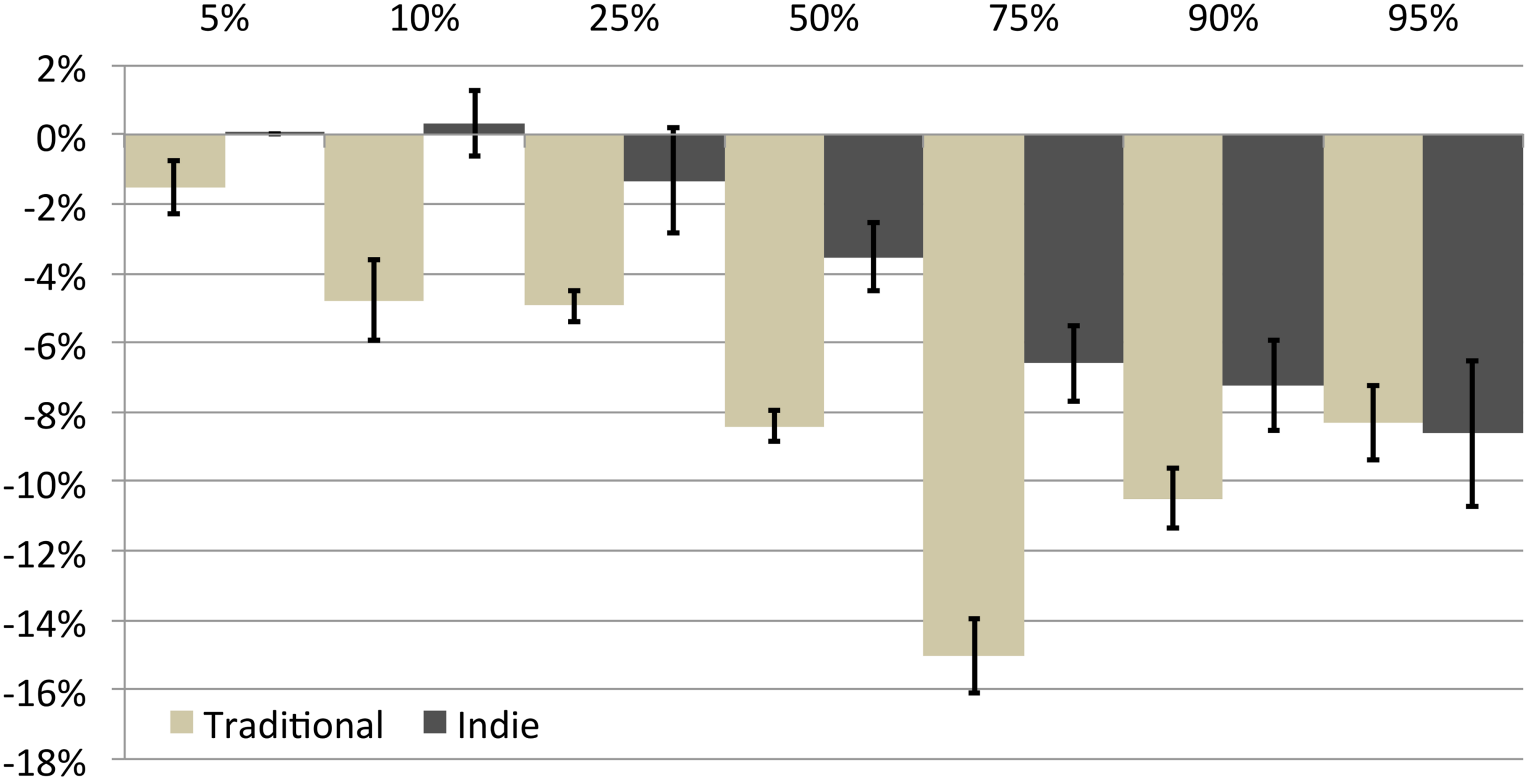
Female price gap by quantile for titles published 2002-2012. On the x-axis, 5% represents the quantile with the lowest prices and 95% the quantile with the highest. The bars measured by the y-axis represent the percent difference in the maximum prices for titles at each quantile.

Using quantile regression, we find a statistically significant female price penalty ranging from 2% to 15% in traditional publishing spanning the price distribution. For indie, the difference in pricing is not significant until the 25th percentile, and then the female price penalty also emerges, ranging from 1% to 9%. Thus, the female price penalty, representing within-job discrimination, also exists in indie publishing but is lower overall, perhaps in part due to the overall lower price points of indie titles. Fig 4 illustrates the price gaps over the distribution of prices.

In sum, as with allocative and valuative discrimination, indie publishing decreases but replicates the within-job discrimination found in traditional publishing. At the same time, the undifferentiated effects of these discrimination mechanisms (Model 1, Table 3) on price are relatively very small for indie compared to traditional publishing, suggesting substantial overall disruption of the impact of these discrimination mechanisms on inequality.

## Discussion

In this paper, we exploit a natural experiment to explore differences in discrimination mechanisms in traditional and indie publishing. The patterns of inequality in book pricing and genre assignment in traditional publishing closely reflect the well known patterns of gender-based wage inequality in the traditional labor market at large, in terms of segregation, valuation of female-dominated fields, and gender-based bias within fields. However, the rise of indie publishing provides an opportunity to examine the difference between inequality patterns in traditional labor markets, where firms dominate, and those in the gig economy, where firm influence diminishes or disappears relative to worker and market influences.

What does the price gap in books teach us about discrimination and inequality in the gig economy? We find evidence of gender discrimination in both traditional and indie publishing. We find greater equality in allocation of authors to genres in indie publishing, but the overall gender distribution of authors appears largely similar when publishers act as gatekeepers or when authors themselves decide how to classify their own books. We also find that female-dominated genres are valued less by publishers and indie authors than male-dominated ones, although these valuative differences are also smaller among indie authors than among traditional publishers. Finally, whether publishers set the price or authors do, titles by authors with distinctly female names are priced lower even within the same genre categories. Within-genre inequality is again more strongly pronounced for titles published by traditional publishers (9% average price gap) than by indie authors (4%), who show more egalitarian pricing especially at the low end of the price distribution. These reduced but replicated patterns of gender discrimination observed in indie publishing compared to traditional publishing suggest that the discriminatory behaviors of firms in traditional publishing have reflected not simply the biases and prejudices of these firms but also those of authors and/or the market. They further suggest that publishing houses have not by and large buffered their authors from a discriminatory market, but may actually have heightened the potential for gender discrimination. Moreover, gender discrimination, though apparent in indie publishing, has a relatively small impact on gender inequality in pricing. Combined, the various types of discrimination lead to a 7% reduction in prices for female authors compared to 45% in traditional publishing. While this substantial disruption in inequality results from the less severe patterns of discrimination in indie publishing, it is also due in large part to the lower prices and smaller range of prices for books in indie publishing.

This paper examined the price or piece rate for books. Average book prices are lower and the range more compressed in indie compared to traditional publishing. However, the lower average prices for indie books do not necessarily reflect lower potential earnings from a sale, as these are also shaped by possible advances (awarded to some but not all traditionally published authors and in varying amounts), royalty structure, and overall level of sales. Indie authors are positioned to retain a greater percentage of income from sales compared to their traditionally published counterparts, but they also bear any costs typically assumed by traditional publishers for producing, distributing, and marketing books. In terms of sales, the increased supply of titles from both traditional publishers and indie authors has not corresponded to similar changes in reading habits and demand [34], such that while some books and authors, especially bestsellers, enjoy voluminous sales, others may see few or none. Thus, while reflective of the price-setting experience for workers in the gig economy, especially those who sell products online, the actual compensation story is far more complex.

This study has several limitations. Author names, our measure of gender, are not necessarily accurate reflections of actual author gender, which may or may not be known to publishers and readers, with potential implications for sorting and decisions about value. However, the decision of what name to put on a book cover, including whether to use a pen name or to use initials instead of names, represents a conscious choice by publishers and authors about how to face the market. It is unclear what the choice of an androgynous name or initials is meant to signal. Our analyses take account of this third category of name but do not explore its implications in depth. While we suspect that more female authors use this option than do male authors, we do not make any assumptions in our research about whose books fall into this category. How and why authors and publishers choose to present gendered or androgynous author names is an interesting topic of future research. The androgynous option is present in some sections of the gig economy but not in others, where workers’ names and even photographs may accompany their sales or service profiles. Another important avenue of research concerns the reaction of consumers to author gender. While this study makes an important contribution to the literature on inequality by differentiating the role of the firm, it could not differentiate worker behavior from market preference. Nor could it show the purchasing behaviors of consumers. Yet, market preferences and their match or mismatch to the behaviors of workers also have implications for gender discrimination and inequality in the gig economy.

Our approach in studying discrimination has been to examine opportunity rather than motivation. We do not try to explain why discrimination occurs, but merely observe its patterns. In studies of the traditional economy, it is very difficult to separate employer behaviors and prejudices from those of job incumbents and the larger social and market context. In comparing the traditional and gig economies, it is possible to separate the behavior of employers from these other factors.

Generalizing from publishing to the broader gig economy, we expect the gig economy will replicate patterns of gender discrimination while also disrupting gender inequality in pricing. While workers have greater freedom that may reduce the traditionally employer-driven pressures to stick to sex-typical jobs or gigs and to devalue women’s work, our findings suggest continued gender-based segmentation and devaluation in the labor market, whether due to social context or to discrimination from the external market. Within jobs and occupations, we also anticipate that female workers will set lower prices than will male workers. However, if greater direct exposure to market pressures drives down prices overall, then gender inequality in price setting is likely to shrink substantially or even to disappear. Some workers will undoubtedly find greater opportunity and prosperity available to them in the gig economy compared to their prospects in the traditional firm-dominated labor market, particularly if they are able to capture a greater volume of sales or gigs. However, others may find greater apparent equality and opportunity but low actual earnings, especially if, as in the case of books, the increase in supply is not matched by a change in market demand for particular goods or services.
